# The Effect of Bilayered Bioactive Coating on Polycaprolactone Electrospun Scaffold Biocompatibility, Bioabsorption and Cellular Properties

**DOI:** 10.3390/polym17212813

**Published:** 2025-10-22

**Authors:** Victor I. Sevastianov, Evgeniy A. Nemets, Alexey M. Grigoriev, Aleksandra D. Belova, Vyacheslav Yu. Belov, Lyudmila A. Kirsanova, Anna S. Ponomareva, Nikita V. Grudinin, Vladimir K. Bogdanov, Alla O. Nikolskaya, Eugenia G. Kuznetsova, Ekaterina A. Guseva, Yulia B. Basok, Sergey V. Gautier

**Affiliations:** 1 The Shumakov National Medical Research Center of Transplantology and Artificial Organs, 123182 Moscow, Russia; viksev@yandex.ru (V.I.S.); bear-38@yandex.ru (A.M.G.); sashak1994@mail.ru (A.D.B.); w.000000000@yandex.ru (V.Y.B.); ludochkakirsanova@mail.ru (L.A.K.); a.s.ponomareva@gmail.com (A.S.P.); zbignev.religa@mail.ru (N.V.G.); bogdanovv@bk.ru (V.K.B.); allanik64@yandex.ru (A.O.N.); kuzeugenia@gmail.com (E.G.K.); ekaterinaa.guseva.2001@mail.ru (E.A.G.); bjb2005@mail.ru (Y.B.B.); priemtranspl@yandex.ru (S.V.G.); 2 The Institute of Biomedical Research and Technology (IBRT), Autonomous Non-Profit Organization, 123557 Moscow, Russia; 3 The Sechenov First Moscow State Medical University, 119435 Moscow, Russia

**Keywords:** electrospinning, scaffold, polycaprolactone, tissue engineering

## Abstract

Bioabsorbable scaffolds from synthetic polyesters are widely used in the field of tissue engineering. However, their hydrophobic surface and lack of suitable functional groups are the main limitations related to cell attachment. The aim of this research was to modify the surface of polycaprolactone (PCL) scaffolds using a bioactive coating containing heparin bound via albumin spacer and platelet lysate over heparin. Porous scaffolds were produced by electrospinning from 10% PCL (*w*/*w*) solution in methylene chloride (25 kV voltage, 100 mm distance between electrodes and 4 mL/h feedrate), which demonstrated 5.5 ± 1.1 MPa Young’s modulus, 2.5 ± 0.4 MPa tensile strength and 321 ± 29% elongation at break. Bioactive coating does not change the structure and mechanical properties of the scaffolds. Treated scaffolds are biocompatible and have no cytotoxic effect in direct contact with cells. Functionalization also promotes the in vitro adhesion and proliferation of human adipose mesenchymal stromal cells. After 7 days of incubation, the PCL scaffold modified with the heparin–platelet lysate complex had a cell density of 185.6 ± 15.7 cells/mm^2^ compared to 79.5 ± 7.8 cells/mm^2^ for nontreated control. The intramuscular implantation of scaffolds revealed that immobilization of heparin alone prolongs the acute phase of the inflammatory reaction. However, subsequent treatment with platelet lysate minimizes the inflammatory reaction, slows the rate of implant absorption, and accelerates vascularization. The results obtained show that the developed bioactive coating improves the cellular properties of PCL electrospun scaffolds and can be used to form in vivo tissue-engineered constructs.

## 1. Introduction

Porous scaffolds produced from biocompatible and bioresorbable materials, capable of mimicking the structural framework properties of the extracellular matrix (ECM), constitute a critical component of tissue-engineered constructs (TECs). Over recent decades, among various techniques for producing highly porous scaffolds—such as a particulate leaching method, phase separation, cryostructuring, and 3D printing [1,2,3,4]—electrospinning has emerged as the most popular method. This preference is attributed to its simplicity in producing porous architectures that closely mimic the native ECM structure of natural tissues [5,6,7]. Currently, electrospinning enables the production of numerous nanofibrous scaffolds from natural polymers, including collagen, gelatin, silk fibroin, and chitosan, as well as bioresorbable synthetic polymers, predominantly polycaprolactone (PCL), polylactic acid (PLA), and its copolymers with glycolic acid [8].

Compared to high-molecular-weight naturally derived compounds, bioresorbable synthetic polyesters exhibit superior mechanical properties, enhanced processability, and reproducibility of key characteristics during production. Furthermore, their properties can be extensively tuned to meet clinical requirements, such as degradation rates and mechanical strength, which is particularly crucial for applications subjected to cyclic mechanical loads; for example, vascular grafts [9]. However, scaffolds composed of synthetic polymers often have highly hydrophobic surfaces and lack bioactive components necessary for promoting cell adhesion, proliferation, and differentiation [5,8,10]. To improve the biological properties of bioresorbable polyester scaffolds, various physical and chemical surface modification techniques have been employed, including plasma treatment using oxygen- or nitrogen-containing gases [11,12,13], as well as hydrolysis and aminolysis processes [14,15,16,17]. These treatments enable broad modulation of surface charge, hydrophilicity, and surface energy by generating polar functional groups (hydroxyl, carboxyl, and amino) on the polymer surface, thereby enhancing cellular interactions.

In addition, these functional groups serve as anchor points for the covalent immobilization of biologically active molecules, facilitating improved cell–material interactions through active stimulation of cell adhesion and proliferation. This can be achieved, for instance, by covalent attachment of protein fragments responsible for adhesive properties [18,19,20,21]. Alternative strategies for enhancing cell adhesion, proliferation, and viability include the physical adsorption or covalent immobilization of growth factors on the polymer surface, as well as their encapsulation within the polymer scaffold [22,23,24]. Passive adsorption often results in rapid, uncontrolled release of biomolecules [25], while covalent immobilization can create a stable bioactive layer [22,26]. Nevertheless, this approach carries the risk of growth factor inactivation due to the involvement of functional groups critical for their biological activity. Conversely, embedding growth factors within the scaffold matrix allows for their gradual release through diffusion, although this method may also pose risks of uncontrolled, burst release. Despite this, it is generally considered safer than covalent bonding since it preserves growth factor activity [22,23].

Particular attention has been given to strategies that preserve the growth factor bioactivity by leveraging auxiliary agents immobilized on scaffold surfaces that possess specific binding sites for growth factors, such as blood plasma components (fibronectin, fibrinogen, fibrin) [23,27] or glycosaminoglycans (e.g., heparin) [28]. Heparin is frequently immobilized on the surfaces of blood-contacting devices to enhance their hemocompatibility [29,30]. Heparin exhibits maximal anticoagulant activity when immobilized at a distance from the surface using a spacer. Common spacers include diaminoalkanes [31], polyethylene oxide [32], polyethyleneimine [33], proteins adsorbed onto polymer surfaces (such as gelatin or albumin) [34,35], as well as protein-heparin conjugates [36,37].

Platelets serve as a source of numerous growth factors, which act as chemoattractants and regulate cell proliferation, maturation, and ECM synthesis. Consequently, platelet lysate is widely used as a growth supplement in cell culture media [38,39,40] and is applied in diverse medical interventions, including wound healing [41], treatment of severe ocular surface disorders [42], enhancement of diabetic foot ulcer repair [43], and as part of biomaterials for engineering various organs and tissues [44,45]. However, to date, the use of platelet lysate as a source of growth factors aimed at improving cell adhesion and proliferation on bioresorbable polyester scaffolds with immobilized heparin remains unexplored. The aim of this research was to modify the surface of the PCL scaffolds using a two-layer bioactive coating containing heparin bound to the polymer surface via an albumin spacer which allows for control of surface-induced blood clotting and platelet lysate over heparin to improve scaffold biocompatibility and cellular properties.

## 2. Materials and Methods

### 2.1. Preparation of Scaffolds

Scaffolds with a thickness of 352 ± 19 µm were produced from 2 mL of a PCL (Mw 80,000, Sigma-Aldrich, St. Louis, MO, USA) solution at concentrations ranging from 8 to 12% (*w*/*w*) in methylene chloride (ECOS-1, Moscow, Russia), following a protocol previously developed for scaffolds fabricated from poly(hydroxybutyrate-co-hydroxyvalerate) [46]. Electrospinning was performed using a NANON-01A apparatus (MECC CO, Fukuoka, Japan) at an applied voltage of 25 kV between the electrodes, feedrate and a 100 mm distance to the collector, employing an 18G needle. Optimization of the electrospinning process to obtain a PCL scaffold with the desired morphology was achieved by varying two parameters: polymer solution concentration ranging from 8 to 12% (*w*/*w*) and solution feedrate ranging from 2 to 6 mL/h. Upon completion of the deposition process, the scaffolds were dried at 37 °C for 2 h, followed by vacuum drying at a residual pressure of 10–20 mmHg and 37 °C for 24 h to remove residual solvent.

### 2.2. Scaffolds Characterizations

#### 2.2.1. Scanning Electron Microscopy

The surface morphology of the scaffolds was examined using a JSM-6360LA scanning electron microscope (JEOL Ltd., Tokyo, Japan) operated at an accelerating voltage of 5 kV. To create a conductive coating, gold was sputter-deposited using a JFC-1600 device (JEOL Ltd., Tokyo, Japan) for 40 s at a constant current of 5–7 mA.

#### 2.2.2. Estimation of Biomechanical Properties of the Samples

Biomechanical testing of the scaffolds was performed using a Shimadzu EZ Test EZ-SX testing machine (Shimadzu Corporation, Kyoto, Japan) equipped with Trapezium X software, version 1.2.6, at a tensile rate of 5 mm/min.

### 2.3. Bioactive Agents Immobilization

Bioactive agents were immobilized following a protocol previously developed [47]. PCL scaffolds were incubated in a solution of bovine serum albumin (Sigma-Aldrich, St. Louis, MO, USA) (1 mg/mL) for 1.5–2 h at 37 °C, followed by treatment with an aqueous heparin solution (Sigma-Aldrich, St. Louis, MO, USA) (Hep, 1 mg/mL) for 1.5–2 h at 37 °C. The coating fixation was performed using a 1% glutaraldehyde solution for 18 h at room temperature, after which the scaffolds were re-treated with the same heparin solution for 1.5–2 h at 37 °C. Between each step the scaffolds were rinsed three times with 100 mL of distilled water. The resulting heparinized scaffold (PCL-Hep) was dried at 37 °C, subsequently vacuum-dried at room temperature under residual pressure of 10–20 mm Hg and sterilized by gamma irradiation at a dose of 1.5 Mrad.

The required volume of human platelet lysate solution was prepared aseptically by reconstituting a lyophilized preparation (Renam, Moscow, Russia) with calcium- and magnesium-free Hanks’ Balanced Salt Solution (HBSS, Gibco, Thermo Fisher Scientific, Waltham, MA, USA). Sterile PCL-Hep samples were treated with the platelet lysate solution under aseptic conditions for 1 h at 37 °C immediately prior to experimentation (PCL-Hep-PL).

Model bioactive coatings were prepared according to the aforementioned methodology on the bottom of the wells of a flat-bottom 24-well culture plate (CELLSTAR, Greiner Bio-One, Frickenhausen, Germany).

### 2.4. Heparin Determination

#### 2.4.1. Surface-Bound Heparin

To determine the amount of immobilized heparin, a spectrophotometric detection method was employed, based on the shift in the absorption maximum of toluidine blue resulting from the dye binding to the charged ionogenic groups (sulfo- and carboxyl groups) of heparin [48]. The quantity of immobilized anticoagulant was determined according to a previously established calibration curve correlating the absorption intensity at λ = 530 nm in a 0.0005% aqueous dye solution with the heparin concentration, which exhibited linearity within the range of 0.1 to 0.8 mg/mL.

#### 2.4.2. Heparin Release

The activated partial thromboplastin time (APTT) assay was employed to investigate the binding strength of heparin to the scaffold surface [49]. The test material was introduced into human platelet-poor plasma (Renam, Moscow, Russia) at a ratio of 0.1 cm^3^/mL and incubated at 37 °C for 60 min. APTT measurement was conducted in accordance with the manufacturer’s instructions (Renam, Moscow, Russia). To prepare the reagent, 2.5 mL of distilled water was added to the vial containing the APTT reagent and the contents were dissolved by gentle agitation. In the measuring cuvette of the Fibrintimer II hemocoagulometer (Dade Behring Inc., Eschborn, Germany), 50 µL of blood plasma or plasma post-incubation with the test material was mixed with 50 µL of the APTT reagent and incubated at 37 °C for 3 min. Subsequently, 50 µL of 0.025 M calcium chloride solution was added, and the coagulation time was recorded. The coagulation time obtained for the plasma incubated with the tested sample was compared to that of the original blood plasma. An increase in coagulation time indicated the release of weakly bound heparin from the surface, the activity of which was quantified based on a previously established calibration curve.

### 2.5. In Vitro Biocompatibility Testing

#### 2.5.1. Surface Induced Hemolysis

The study was conducted in accordance with the methodology recommended by ISO 10993-4:2017 Biological evaluation of medical devices [50]: for diluted suspensions of erythrocytes in contact with test materials, hemolysis is reported as a percentage of hemoglobin which has been released into the supernatant normalized to the total hemoglobin present at the start of the test [i.e., (free hemoglobin concentration/total hemoglobin concentration) × 100%]. Complete erythrocyte destruction corresponds to 100% hemolysis. Extracts from the samples were prepared by incubating 1 g of a scaffold in 30 mL of 0.9% sodium chloride solution at 37 °C for 120 min. Distilled water was used as a negative control (corresponding to 100% hemolysis). Subsequently, 200 µL of citrated rabbit blood (1:9 dilution) was added to a test tube containing 10 mL of the extract or control, gently mixed, and incubated at 37 °C for 1 h. Following centrifugation at 2000 rpm for 20 min, the optical density of the supernatant was measured at a wavelength of 545 nm using a Tecan Spark 10M plate reader (Tecan Trading, Männedorf, Switzerland). A sample is considered hemocompatible if percentage of hemoglobin ≤ 2%.

Rabbit blood was taken from New Zealand White rabbits (males). The manipulations did not cause pain to the animals and were carried out in compliance with the Russian legislation: GOST 33215-2014 (Guidelines for accommodation and care of laboratory animals. Rules for equipment of premises and organization of procedures) [51] and GOST 33216-2014 (Guidelines for accommodation and care of laboratory animals. Rules for the accommodation and care of laboratory rodents and rabbits) [52]. The work was approved by the Local Ethics Committee at the Shumakov National Medical Research Center of Transplantology and Artificial Organs, Moscow, Russia (24 January 2020, Protocol No. 240120-1/1e).

#### 2.5.2. Complement Activation

To obtain serum, rabbit blood was collected into tubes without anticoagulants and allowed to stand at room temperature for 2 h, followed by centrifugation at 1000× *g* for 20 min. The test material was added to the rabbit serum at a ratio of 0.1 cm^3^/mL and incubated at 37 °C for 60 min.

Using the concentrated standard solution and solvent provided in the reagent kit from Renam (Moscow, Russia), standard solutions of the complement component C3a were prepared in a concentration range from 0.75 to 50 ng/mL. The solvent used for preparing the standard solutions served as the zero calibration point.

Subsequent analyses were conducted according to the manufacturer’s instructions.

### 2.6. Cytotoxicity Assay

The determination of the cytotoxicity of scaffold samples in vitro was evaluated in accordance with ISO 10993-5:2009 [53] on a NIH/3T3 mouse fibroblast culture (ATCCRCRL-1658, American Type Culture Collection, Manassas, VA, USA) by direct contact.

Fibroblasts were cultured in standard culture vials with an area of 25 cm^2^ (CELLSTAR Greiner Bio-One, Frickenhausen, Germany) in a complete growth medium containing DMEM (Dulbecco’s modified Eagle medium) with a high glucose content (4.5 g/L, DMEM high glucose with HEPES, PanEco, Moscow, Russia), 10% calf serum (Biosera, Cholet, France), 1% antibiotic–antimycotics Anti-Anti (Gibco, Thermo Fisher Scientific, Waltham, MA, USA) and 2 mM glutamine (PanEco, Moscow, Russia) in a CO_2_ incubator under standard conditions (37 °C, humidified atmosphere containing (5 ± 1)% CO_2_). The cells were removed from the cultured plastic surface using the dissociating reagent TrypLE Express Enzyme (Gibco, Thermo Fisher Scientific, Waltham, MA, USA). The initial number of cells in the suspension was determined using the TC20TM Automated Cell Counter (Bio-Rad Laboratories Inc., Hercules, CA, USA). To study the cytotoxic effect, fibroblasts were seeded into 96-well flat-bottomed culture plates (CELLSTAR Greiner Bio-One, Frickenhausen, Germany) at a concentration of 1–2 × 10^4^ cells per well and incubated in a complete growth medium until the formation (80 ± 10) % of a monolayer under standard conditions. Then, the test (*n* = 8) and control samples were introduced into wells with a cellular monolayer and incubated for 24 h. Fibroblast culture was visually assessed using a Nikon Eclipse TS 100 microscope (Nikon, Tokyo, Japan).

A DMEM nutrient medium with a high (4.5 g/L) glucose content (PanEco, Moscow, Russia) was used as a negative control sample to demonstrate the background cell reaction. As a positive control sample to demonstrate the appropriate reaction of the test system, the single-element aqueous zinc standard 10,000 mcg/mL (Sigma-Aldrich, St. Louis, MO, USA) was used.

### 2.7. In Vitro Cell Cultures

#### 2.7.1. Cells Source

Adipose tissue samples were obtained with the informed consent of living healthy donors during liver transplantation under general anesthesia. The study was conducted in accordance with the guidelines of the Helsinki Declaration and approved by the Local Ethics Committee at the Shumakov National Medical Research Center of Transplantology and Artificial Organs, Moscow, Russia (15 November 2019, Protocol No. 151119-1/1e). The culture of mesenchymal stromal cells (MSCs) isolated from human adipose tissue was established at the Shumakov National Medical Research Center of Transplantology and Artificial Organs, according to a previously developed protocol [54]. Following thawing, MSCs were seeded into standard culture flasks with a growth surface area of 25 cm^2^ (CELLSTAR Greiner Bio-One, Frickenhausen, Germany) and cultured in complete growth medium DMEM/F12 (PanEco, Moscow, Russia) supplemented with 10% fetal bovine serum (HyClone, Logan, UT, USA), 10 μg/mL human basic fibroblast growth factor (FGF-2, Peprotech, AF-100-18B, Rocky Hill, NJ, USA), the antibiotic and antifungal solution Anti-Anti (Gibco, Thermo Fisher Scientific, Waltham, MA, USA), 1 mM HEPES buffer (Gibco, Thermo Fisher Scientific, Waltham, MA, USA), and 2 mM alanyl-glutamine (PanEco, Moscow, Russia). Cultures were maintained in a CO_2_ incubator under standard conditions: 37 °C, in a humidified atmosphere containing (5 ± 1) % CO_2_. MSCs at passage III were employed in the experiments.

Prior to the experiment, cells were detached from the culture plastic using the dissociating reagent TrypLE Express Enzyme (Gibco, Thermo Fisher Scientific, Waltham, MA, USA) and prepared as suspensions at the required cell concentrations. The initial cell number in the suspension was determined using an automated cell counter (TC20 Automated Cell Counter, BIORAD, Singapore), concurrently assessing viability through the exclusion of the trypan blue dye (BIORAD, #145-0013, Singapore).

#### 2.7.2. Cell Seeding

For the model coatings, the initial seeding density of MSCs was 3 × 10^3^ cells/cm^2^. The initial cell seeding density on scaffold surfaces (discs with a diameter of 8 mm) was 5 × 10^4^ cells/cm^2^. Cells were allowed to adsorb onto the scaffold surfaces for 2 h, after which the discs were carefully transferred into 50 mL tubes containing 5 mL of complete growth medium. Subsequently, the samples were incubated under standard conditions in a CO_2_ incubator. Scaffolds made of PCL and wells of tissue culture plastic without coating were used as control samples.

#### 2.7.3. Cell Staining

The distribution pattern of cells across the sample surface, cell viability (expressed as the percentage of living cells relative to the total cell number), morphology, and proliferative activity after 1 day and 7 days of incubation were evaluated using live-cell fluorescence microscopy. The assessment employed the Live/Dead Viability/Cytotoxicity Kit (Molecular Probes by Life Technologies, Waltham, MA, USA) in accordance with the manufacturer’s recommended protocol.

### 2.8. Scaffolds Local Effect and Absorption Testing In Vivo Study

#### 2.8.1. Scaffold Implantation

The Local Ethics Committee at the Shumakov National Medical Research Center of Transplantology and Artificial Organs (Moscow, Russia) approved this study (12 December 2022, Protocol No. 22.12.22-1-7e). Experiments were performed using 36 Wistar 6-month-old female rats, weighing 350–380 g (Krolinfo, Orekhovo-Zuyevo, Russia). The implantation was carried out under anesthesia (Zoletil100, Virbac, Carros, France, 15 mg/kg). Manipulations did not cause pain to the animals and were carried out in compliance with Russian legislation: GOST 33215-2014 (Guidelines for accommodation and care of laboratory animals. Rules for equipment of premises and organization of procedures) and GOST 33216-2014 (Guidelines for accommodation and care of laboratory animals. Rules for the accommodation and care of laboratory rodents and rabbits). In compliance with the rules of asepsis and antiseptics, after shaving and disinfecting the right paw, a lateral skin incision was made using blunt dissection. About 50 mg of the scaffold was put into the intramuscular pocket in the quadriceps muscle. The muscle and skin were sutured in layers using absorbable sutures (Vicryl 6-0, Ethicon, Issy-les-Moulineaux, France). After surgery, no antibiotic treatment was administered. Animals were sacrificed 4 weeks, 8 weeks, 12 weeks and 24 weeks after surgery. The implants were harvested and fixed in 10% neutral formalin (BioVitrum, Moscow, Russia) for 7 days. Samples were then dehydrated and embedded in paraffin. Thin sections (approx. 4–5 μm) were made in the middle of the sample using microtome (Leica RM3255, Leica, Wetzlar, Germany). Hematoxylin and eosin (H&E) staining was performed on sample slices.

#### 2.8.2. Histological Investigation

For histological analysis, the implanted materials were explanted together with the surrounding tissues at predetermined time points post-surgery. Samples were fixed in 10% formalin solution buffered with phosphate, followed by standard dehydration through the ascending concentrations of ethanol. Subsequently, tissues were immersed sequentially in ethanol-chloroform mixtures and chloroform alone before embedding in paraffin.

Sections 5–6 µm-thick were obtained from paraffin blocks using a Leica RM3255 microtome. The sections were then deparaffinized, rehydrated, and stained with hematoxylin and eosin (BioVitrum, Moscow, Russia) to provide a general overview of the tissue structure. Additionally, Masson’s trichrome staining (BioVitrum, Moscow, Russia) was performed to detect overall collagen distribution.

The microscopic examination and photodocumentation of the prepared slides were carried out using a Nikon Eclipse 50i microscope (Nikon, Tokyo, Japan) equipped with a digital camera.

A comparative evaluation of the local tissue response to the implantation of the modified samples (PCL-Hep and PCL-Hep-PL) versus the control (untreated PCL) was conducted. Parameters assessed included the degree of cellular reaction, absorption, vascularization, and formation of the connective tissue capsule.

### 2.9. VEGF Adsorption/Desorption Determination

A heparinized scaffold (0.2 cm^3^) was incubated in 1 mL of platelet lysate at 37 °C for 60 min. Thereafter, the vascular endothelial growth factor (VEGF) concentration in the plasma before and after contact with the scaffold was measured using an ELISA kit (Vector Best JSC, Novosibirsk, Russia) according to the manufacturer’s instructions. The difference between the two values was used to calculate the amount of surface bound VEGF (pg/cm^3^) by multiplying the obtained value by 5. Desorption of scaffold bound VEGF was assessed after three washes of the platelet lysate loaded scaffolds with a 10× volume of DMEM/F12 (PanEco, Moscow, Russia), followed by incubation in 1 mL of DMEM/F12 for 1 and 7 days. The time points were selected to match the intervals typically used for evaluating cell adhesion and proliferation in vitro.

### 2.10. Statistical Analysis

Data were analyzed with SPSS26.0 statistical software. The distribution of variables was tested with the Shapiro–Wilk procedure. The results were compared using the Mann–Whitney unpaired *t*-test and Kruskal–Wallis test, where *p* < 0.05 was considered statistically significant.

## 3. Results and Discussion

### 3.1. Morphology and Mechanical Characterization

In the development of scaffolds for tissue engineering, pore size is one of the critical parameters. By adjusting pore size, it is possible to provide optimal conditions not only for nutrient transport and metabolic waste removal, but also to enhance cell viability, proliferation, and differentiation, as well as to facilitate cell migration into the scaffold volume. The optimal pore size is specific to each cell type [55,56,57].

Fiber diameter also plays an important role in the fabrication of tissue-engineered constructs (TECs), as scaffold fibers should mimic the fibrillar proteins that are part of the ECM. Since pore size is closely related to the diameter of the fibers forming the porous scaffold (the thicker the fibers, the larger the pore diameter), the small pore size and poor pore interconnectivity of nanofiber scaffolds often lead to insufficient cell penetration, which constrains their applications [58]. To overcome this problem, additional efforts are needed to create macrochannels in such scaffolds that can significantly facilitate cell penetration [59,60].

Therefore, in order to produce PCL scaffolds with fiber diameters in the micrometer range and pore sizes exceeding 10 μm, we optimized our previously developed technology for fabricating scaffolds based on poly(hydroxybutyrate-co-hydroxyvalerate) [46]. The main drawback of that earlier approach was the high Young’s modulus (~50 MPa), which necessitated the incorporation of large amounts of gelatin (up to 200% by weight of the polymer) to reduce stiffness. This, however, resulted in deterioration of the other mechanical properties.

The primary factors influencing the diameter of electrospun fibers include polymer concentration, solution conductivity, electric field strength, and feedrate. Since, in certain cases, increasing the electric field strength either has no effect on fiber diameter [61] or can produce opposite trends—either increasing [62] or even decreasing it [63]—polymer concentration and feedrate were selected as the parameters for optimization in this study.

**Figure 1:**
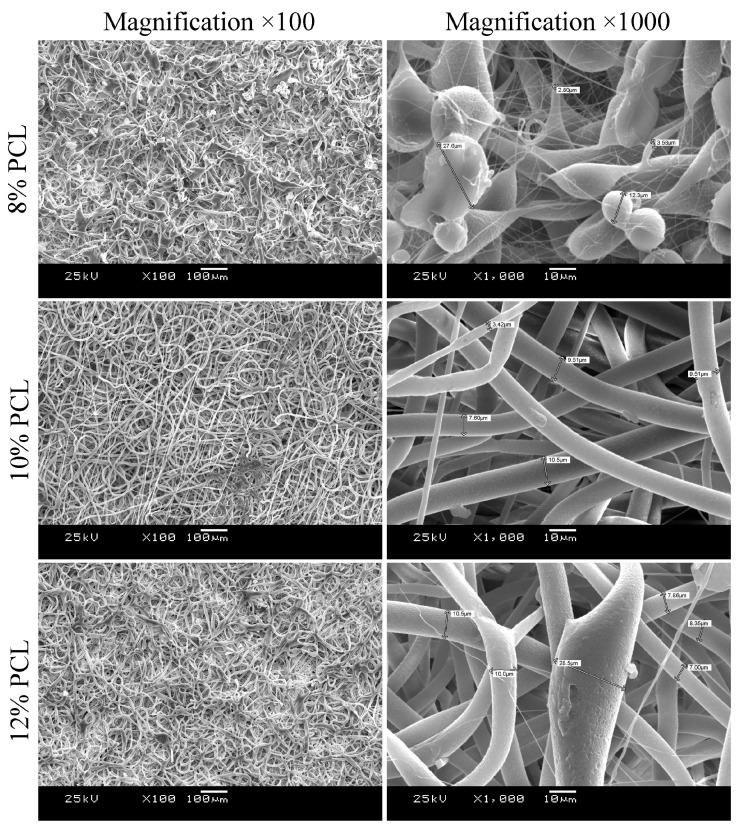
The effect of the concentration of PCL solution on the structure of scaffolds, feedrate 4 mL/h.

The effects of polymer solution concentration on the scaffold surface morphology are presented in Figure 1. The processing parameters previously identified as optimal for poly(hydroxybutyrate-co-hydroxyvalerate) (polymer concentration of 8% and a feedrate of 4 mL/h) yielded, in the case of PCL, scaffolds predominantly composed of spherical structures. Droplet formation during electrospinning arises from the interplay of several competing forces. Surface tension promotes minimization of the surface area, driving the jet toward one or more spherical droplets via Rayleigh instability [64], whereas electrostatic repulsion on the Taylor cone surface increases surface area, favoring fine jet formation. Viscoelastic forces resist rapid deformation, supporting fiber formation [64]. Continuous fiber production thus requires a delicate balance between Rayleigh instability and surface tension. Increasing solution viscosity by raising polymer concentration counteracts jet breakup, allowing the jet to persist longer before disintegration into droplets. A continuous, bead-free fibrous structure is achieved only above a critical polymer concentration [65] which, for PCL, was higher than for higher-molecular-weight poly(hydroxybutyrate-co-hydroxyvalerate).

Increasing the PCL concentration to 10% resulted in scaffolds composed mainly of fibers with diameters of 7–10 µm and pore sizes ranging from 10 to 50 µm (Figure 1), dimensions sufficient to facilitate cell migration deep into the scaffold [66,67]. A further increase to 12% led to fiber fusion and spindle-like thickenings, likely due to incomplete solvent evaporation and insufficient fiber stretching before reaching the collector. Thus, scaffolds from the 10% solution exhibited the most favorable morphology and more organized surface architecture compared to those from the 12% solution.

**Figure 2:**
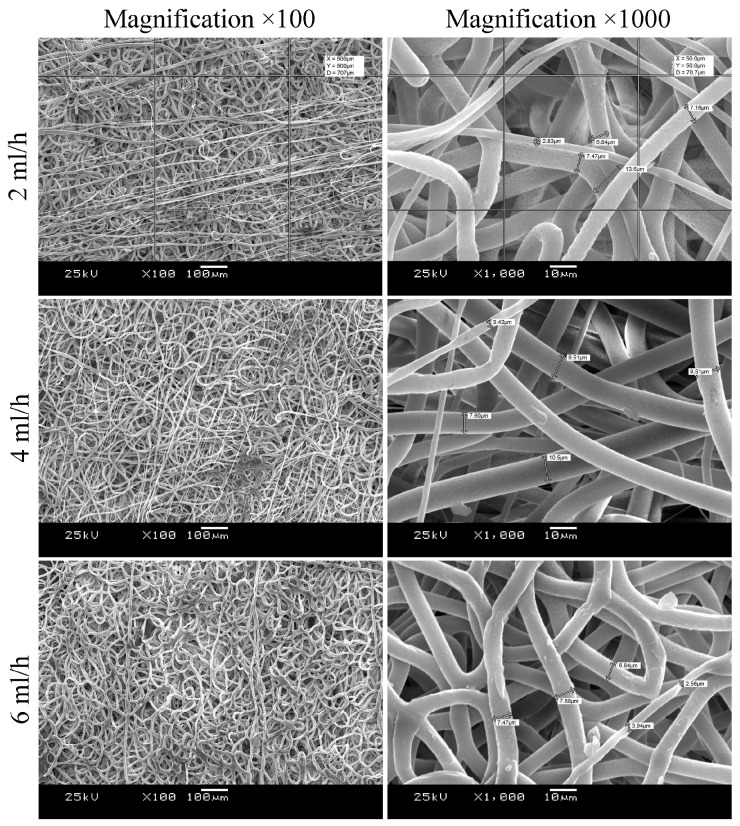
The effect of the feedrate on the structure of scaffolds, 10% PCL solution.

Feedrate also plays an important role in determining fiber morphology. Lower flow rates are generally preferable, as they allow sufficient time for solvent evaporation, producing smooth and uniform fibers [65]. Reducing the feedrate from 4 to 2 mL/h produced no major in scaffold architecture changes (Figure 2), except for a higher proportion of thinner fibers and a slight reduction in pore size. Conversely, increasing the feedrate to 6 mL/h did not significantly affect fiber diameter or pore size but led to fiber fusion, likely due to incomplete solvent evaporation under the given voltage and electrode spacing. All three scaffold variants displayed architectures suitable for tissue engineering applications; thus, the final selection was based on their mechanical properties.

As summarized in Table 1, replacing poly(hydroxybutyrate-co-hydroxyvalerate) with PCL reduced the Young’s modulus by an order of magnitude, bringing it closer to values characteristic of native tissues. Rat aorta was chosen as the reference tissue, as the ultimate goal of this work is the development of small-diameter vascular grafts. The mechanical properties were significantly improved with an increase in feedrate from 2 to 4 mL/min. Samples obtained using feedrate 4 and 6 mL/hour differed little from each other, but the tensile strength value of the scaffold obtained using feedrate 4 mL/hour was closer to the rat aorta. For this reason, a feedrate of 4 mL/min was selected as optimal and used in subsequent experiments.

The application of a biologically active coating onto the scaffold fiber surface is conducted in two stages: first, immobilization of heparin via albumin spacer (PCL-Hep) is performed. Subsequently, the heparinized surface is exposed to platelet lysate (PCL-Hep-PL), resulting in the immobilization of growth factors.

Beyond its anticoagulant properties, heparin can stably bind numerous growth factors that possess specific heparin-binding domains [28,68], including the vascular endothelial growth factor (VEGF), a critical mediator of angiogenesis [68]. The concentration of growth factors on the surface through interaction with heparin immobilized on the scaffold not only significantly enhances scaffold-cell interactions [69], including in vivo [70], but also increases growth factor stability against denaturation and enzymatic degradation under physiological conditions, thereby prolonging their biological activity [44,71]. Furthermore, it has been demonstrated that the presence of plasma proteins does not inhibit the interaction of growth factors with heparinized surfaces [45]. Thus, platelet lysate may serve as a source of growth factors, facilitating improved interactions between heparinized scaffolds and cells through the concentration of growth factors on their surfaces.

The release of immobilized heparin was evaluated using the APTT assay. Upon contact with PCL-Hep-PL, the clotting time of human plasma was 23.0 ± 1.2 s, which did not differ significantly from the baseline value of 21.7 ± 0.8 s observed in untreated serum. This indicates that the heparinized scaffold does not release loosely bound heparin during the procedure.

**Figure 3:**
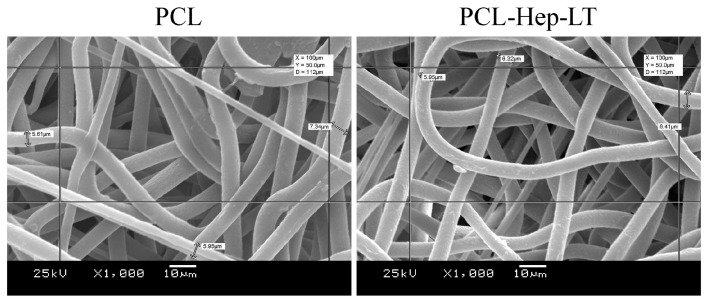
The effect of applying a bioactive coating on the structure of the scaffolds (10% PCL solution, feedrate 4 mL/min).

Importantly, the risk of adverse alterations to the scaffold architecture is minimal, as the bioactive coating is applied via incubation of components in aqueous solutions at physiological temperatures. Indeed, the immobilization of heparin, achieving a surface concentration of 52 ± 6 μg/cm^3^, followed by treatment with platelet lysate, has negligible impact on the PCL fiber surface structure (Figure 3).

The application of the coating resulted in a 1.5-fold reduction in tensile strength and a 15% decrease in maximum elongation, which can most likely be attributed to the hydrophilization of the originally hydrophobic surface of PCL fibers (Table 2).

### 3.2. In Vitro Scaffolds Biocompatibility

To investigate the biocompatible properties of PCL-based scaffolds, we examined the complement system activation, erythrocyte hemolysis, and cytotoxicity induced by contact with the modified samples.

The concentration of complement component C3a after contact with the scaffolds was 2.3 ± 1.5 ng/mL, which did not differ significantly from the baseline human serum value of 2.6 ± 1.6 ng/mL. Hemolysis induced by contact with the PCL-Hep-PL surface was measured at 0.11 ± 0.02%, well below the established acceptance criterion of less than 2%.

**Figure 4:**
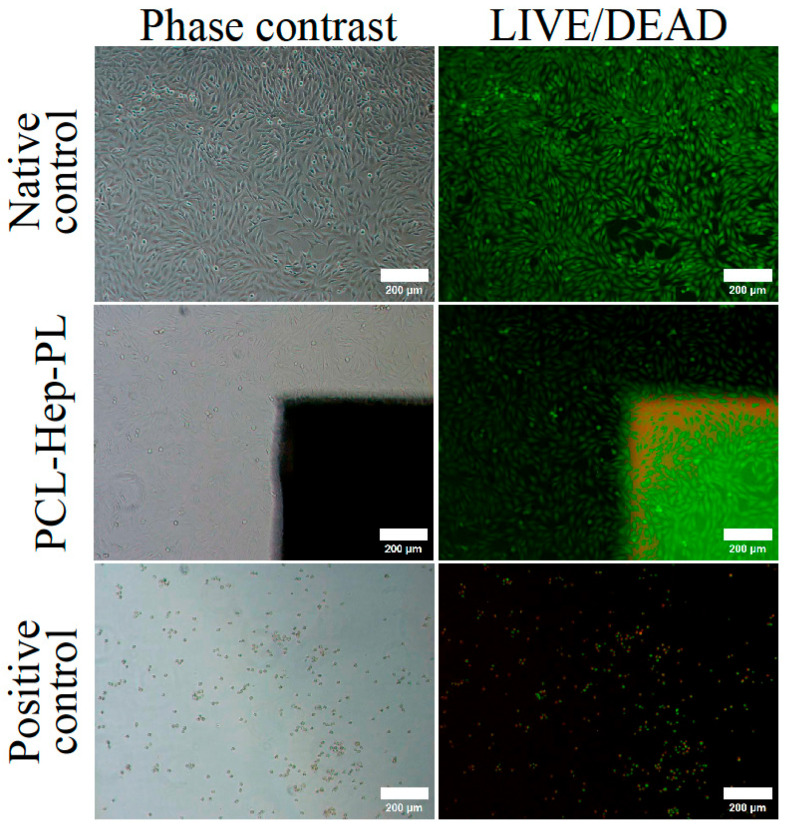
PCL-Hep-PL scaffold cytotoxicity study in vitro. Negative control—DMEM nutrient medium with high (4.5 g/L) glucose content; Positive control—single-element aqueous zinc standard. Scale bar = 100 µm.

In cytotoxicity assays, scaffolds bearing the bioactive coating exhibited no cytotoxic effects: no cellular reactions were observed either adjacent to or beneath the samples (Figure 4).

The cell monolayer appeared visually indistinguishable from the negative control sample, while the positive control displayed extensive cellular debris.

In summary, PCL-Hep-PL scaffolds demonstrate biocompatibility and are therefore suitable for TEC formation.

### 3.3. Effect of the Treatment on the In Vitro Cells-Surface Interaction

To investigate the effect of applying coating on the interaction between PCL scaffolds and cells, MSCs were selected due to the absence of ethical concerns associated with their use [72]. MSCs are widely utilized for the treatment of a broad range of pathologies, including cardiovascular, neurodegenerative, autoimmune diseases, as well as lung, liver, kidney, and orthopedic disorders [73,74,75,76,77,78,79]. In particular, adipose-derived MSCs possess the capability for directed differentiation not only into adipose tissue but also into various mesenchymal tissues, including cartilage and bone [80].

#### 3.3.1. Model Surface

**Figure 5:**
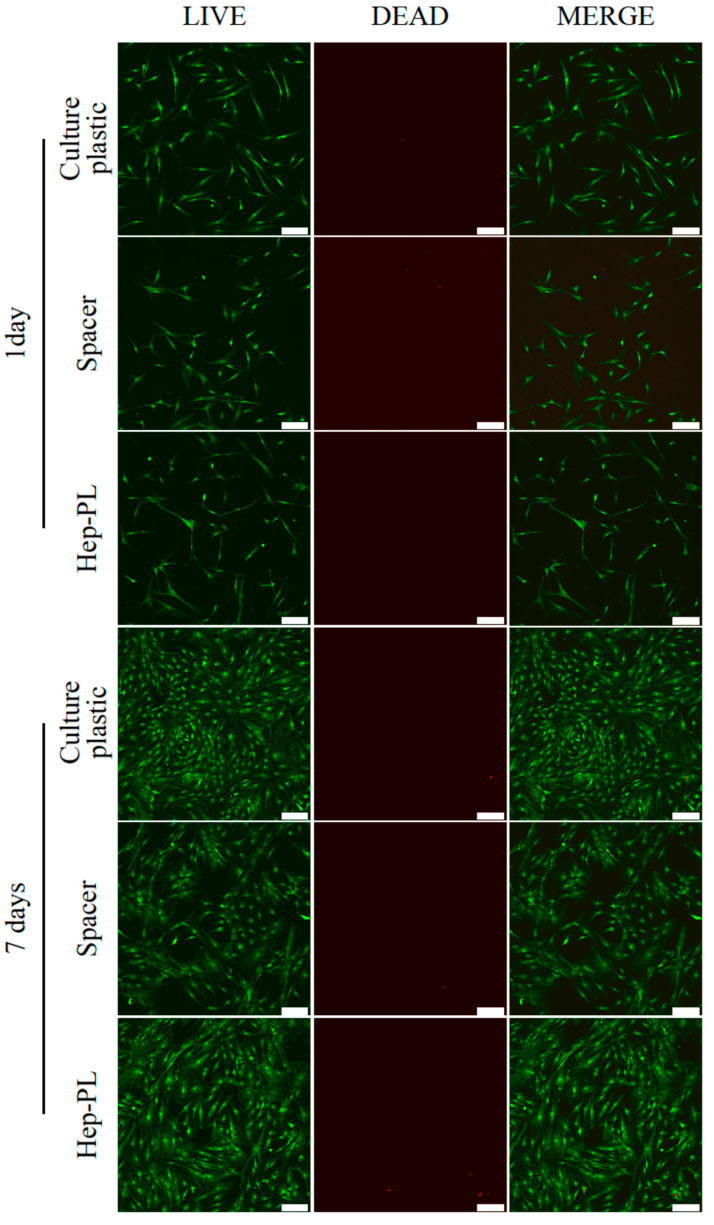
Live/Dead assay performed on MSCs using culture plastic, spacer and Hep-PL bioactive coating over spacer. Fluorescence images of live cells (green), dead cells (red) and merge (green/red). Scale bar 100 µm.

Since, besides the presence of signaling molecules, scaffold characteristics such as surface hydrophilicity and roughness [81], as well as fiber diameter and packing density [82], play significant roles in cell adhesion and proliferation processes, the initial evaluation of the bioactive coating effectiveness was conducted using model coatings formed on the surface of cell culture plates (Figure 5). The positive control was the surface of standard culture plastic, while the negative control corresponded to culture plastic coated with a spacer.

Within the first 24 h, cells have attached to the surface of all samples and spread out (Figure 5). The application of the spacer onto the culture plastic surface resulted in a decrease in the number of viable cells from 13.4 ± 2.4 to 8.2 ± 1.6 cells/mm^2^. The subsequent treatment of the spacer layer with the Hep-PL coating led to a marked increase in adherent cell number (11.3 ± 1.2 cells/mm^2^), although this remained lower than the cell count on uncoated culture plastic. After seven days, the cell numbers on the surfaces of culture plastic, spacer, and Hep-PL coating samples significantly increased to 67.2 ± 11.1, 38.3 ± 3.8, and 49.0 ± 3.9 cells/mm^2^, respectively. At all time points and across all samples, the proportion of viable (green-stained) cells surpassed that of dead cells (red-stained), which did not exceed 1.8 ± 0.3 cells/mm^2^. Thus, the number of viable cells on the Hep-PL coated surface was higher than that on the spacer-treated surface during both the adhesion phase (Day 1) and the proliferation phase (Day 7).

#### 3.3.2. Scaffold Surface

**Figure 6:**
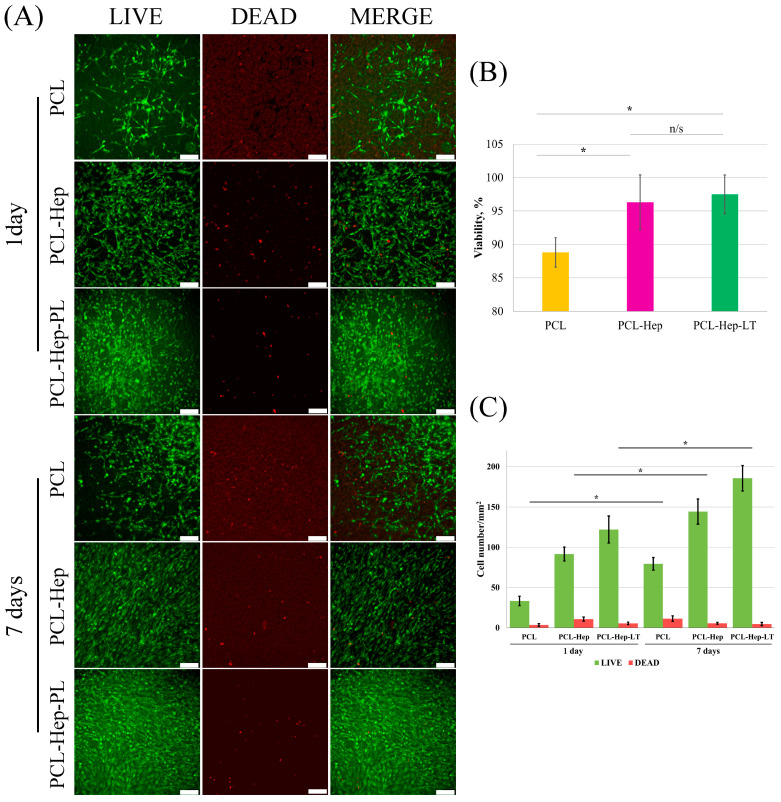
Live/Dead assay performed on MSCs using PCL noncoated control, PCL-Hep and PCL-Hep-PL. (**A**). Fluorescence images of live cells (green), dead cells (red) and merge (green/red). (**B**). Quantitative analysis of all groups. (**C**). Cells viability after 7 days in all groups Data shown as a mean ± SEM of *n* = 9 fields of view with n.s., no significant differences, * *p* < 0.05. Scale bar 100 µm.

Next, the ability of PCL-Hep-PL samples to support MSC adhesion and proliferation was investigated in comparison with unmodified PCL control (Figure 6).

As shown in Figure 6A, adhesion and spreading of viable cells on the surface were observed in TECs composed of PCL, as well as those with PCL-Hep and PCL-Hep-PL modifications. Heparin immobilization significantly increased the number of adherent cells from 33.4 ± 7.8 to 91.6 ± 8.6 cells/mm^2^ (Figure 6C). The application of platelet lysate over the immobilized heparin further enhanced the density of viable cells on the modified PCL surface, reaching 122.1 ± 16.8 cells/mm^2^. By day seven, cell numbers continued to rise (Figure 6C), with cells forming confluent layers on the surface of the modified scaffold. Cell viability was improved as a result of surface modification of PCL fibers with either heparin alone or with the heparin–platelet lysate complex (Figure 6B); however, the highest number of viable cells was observed on PCL-Hep-PL surfaces (185.6 ± 15.7 cells/mm^2^) (Figure 6C). These results indicate that the PCL coated with platelet lysate via heparin immobilization enhances both adhesion and proliferation of the MSCs.

### 3.4. Scaffold In Vivo Biocompatibility and Absorption

A histological analysis of PCL samples revealed specific characteristics at various time points of the experiment.

**Figure 7:**
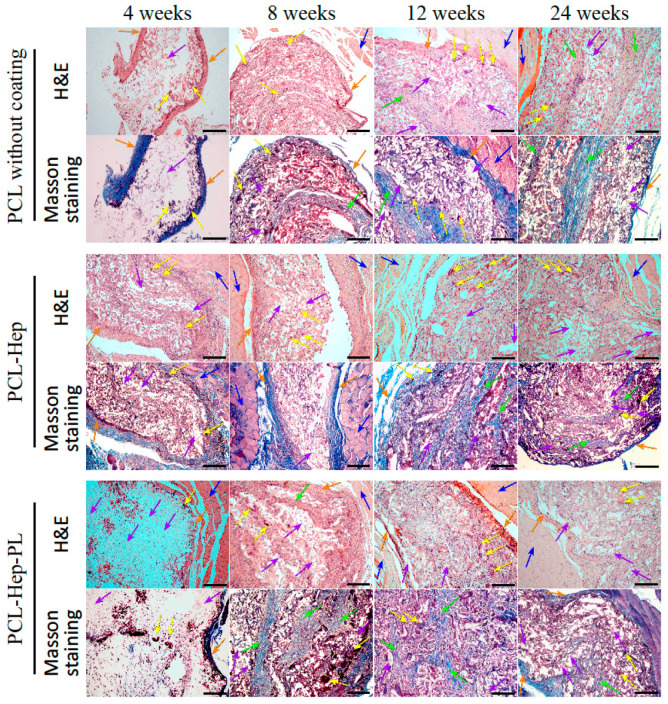
Histological picture of scaffolds after intramuscular implantation. Scale bar 100 µm. Orange arrow—connective tissue capsule; yellow arrow—foreign body giant cells; (polynuclear giant foreign body cells); green arrow—connective tissue strands; blue arrow—muscle tissue; purple arrow—scaffold.

The histological profile of the control sample (uncoated PCL) at 4 weeks (Figure 7) was characterized by the presence of a dense fibrous capsule surrounding the implant perimeter.

Masson’s trichrome staining highlighted collagen fibers within this capsule. At the interface of the inner capsule surface, a moderate macrophage response to the implant material was observed, accompanied by the formation of foreign body giant cells (FBGCs), indicative of phagocytosis and signaling the onset of absorption of the implanted sample (Figure 7). Only isolated FBGCs were detected within the implant, with the majority of the implanted material remaining intact.

At 8 weeks post-implantation (Figure 7), the inflammatory response remains predominantly macrophage-mediated; however, it becomes more pronounced. A subtotal replacement of the implant by foreign body granulomas is observed, indicating active absorption. In addition, connective tissue strands are detected within the implant, partially replacing the implant material with fibrous tissue.

At 12 weeks (Figure 7), giant cell–mediated absorption of the implant continues, with minimal residual intact implant detectable. Well-developed connective tissue strands contain dilated capillaries, a sign of active vascularization. The fibrous capsule surrounding the implant exhibits irregular thickness and is penetrated by thin-walled capillaries filled with erythrocytes.

At 24 weeks (Figure 7), there was a marked reduction in the giant cell reaction, suggesting a lessened overall inflammatory response. The replacement of the implant material by well-vascularized fibrous connective tissue strands became more prominent compared to earlier time points. No visible signs of inflammation were detected in the adjacent muscle tissue.

The histological evaluation of the PCL-Hep scaffold sample (Figure 7) at 4 weeks post-implantation revealed the presence of a thick fibrous capsule enveloping the implant perimeter. This capsule was intermittently penetrated by engorged capillaries. At the outer boundary of the capsule, foci of lymphoid cell infiltration mixed with polymorphonuclear leukocytes were identified, likely reflecting a prolonged acute phase of the inflammatory response. Within the implant itself, a marked macrophage reaction with abundant FBGCs was observed, indicative of active phagocytosis and absorption of the scaffold material. At 8 weeks (Figure 7), the giant cell reaction persisted. Small connective tissue strands appeared within the bulk of the sample, potentially representing early signs of fibrous tissue replacement. By 12 weeks (Figure 7), the trend toward partial substitution by fibrous tissue intensified: the implant mass contained robust fibrous strands incorporating engorged capillaries. Concurrently, absorption by FBGCs appeared more pronounced compared to the earlier time point. The implant was delineated from adjacent tissue by a thin, dense capsule. This clearly demarcated capsule surrounding the implant persisted for up to 24 weeks (Figure 7). Macrophage-mediated absorption continued; however, the FBGC pool within the scaffold decreased, suggestive of an overall attenuation of the inflammatory response. Partial replacement by fibrous tissue, evidenced by an increased number of fibrous strands, was substantially greater than at 12 weeks (Figure 7).

In contrast to the control sample (PCL without coating), where the cellular reaction to the implant at 4 weeks was relatively mild, the experimental PCL-Hep scaffold demonstrated a pronounced macrophage response at the same interval, although signs of an unresolved acute inflammatory phase remained. Notably, encapsulation, neovascularization, partial fibrous tissue replacement, and reduction in the giant cell reaction (Figure 7) occurred at similar time points in both experimental and control specimens. No discernible pathological changes were detected in the adjacent muscle tissue.

The histological profile of the PCL-Hep-PL scaffold (Figure 7) at 4 weeks post-implantation was characterized by a thin capsule encircling the implant. The cellular reaction was mild, with sparse macrophage and giant cell infiltration at the inner capsule boundary. Within the scaffold bulk, only isolated FBGCs were present, and the majority of the implant material appeared intact. Early formation of small thin-walled blood vessels within both superficial and deeper implant zones indicated scaffold vascularization at an early stage. At 8 weeks (Figure 7), the histological pattern changed markedly, showing an active macrophage response with numerous FBGCs and the substantial absorption of the implant. The proportion of preserved implant material markedly decreased. It is important to highlight that the degree of absorption at this time point was the most pronounced among all examined samples. Moreover, fibrous tissue replacement, driven by active growth of connective tissue strands within the scaffold, was more extensive. By 12 weeks (Figure 7), absorption persisted but showed reduced intensity, possibly reflecting a diminished FBGC population. The resorbed implant was replaced by robust connective tissue strands containing engorged capillaries. At 24 weeks (Figure 7), giant cell-mediated absorption and subsequent fibrous tissue substitution continued, with the implant surrounded by an unevenly thick capsule.

Similarly to the control (PCL without coating), the cellular response at early time points (4 weeks) was moderate. However, a distinctive characteristic of the experimental scaffold at this stage was the presence of initial signs of vascularization, whereas vascularization in the control was evident only at 12 weeks. Additionally, a reduction in the FBGC population and corresponding attenuation of the inflammatory response were observed by 12 weeks in the experimental sample, in contrast to a later occurrence in the control. Throughout all implantation intervals, the adjacent muscle tissue in PCL-Hep-PL scaffold recipients remained free of inflammation and fibrosis.

The experimental data obtained allow us to conclude that immobilization of heparin alone prolongs the acute phase of the inflammatory response. The application of platelet lysate onto immobilized heparin accelerates the process of vascularization (Figure 7), presumably due to the angiogenic growth factors contained within the platelet lysate [68]. Consequently, the acute phase of inflammation resolves more rapidly, and the overall inflammatory response tends to subside progressively within 8 to 24 weeks. It is noteworthy that during the early (4–8 weeks) stages of scaffold resorption in PCL-Hep-PL (Figure 7), the material remains largely intact. Presumably, the application of lysate masks the surface and renders the scaffold less accessible to the pool of inflammatory cells, suggesting enhanced implant stability over an extended period.

## 4. Conclusions

The experimental results obtained allow us to conclude that treated scaffolds are biocompatible and have no cytotoxic effect when in direct contact with cells, and the bioactive coating does not change the structure and mechanical properties of the scaffolds and promotes the in vitro adhesion and proliferation of human adipose MSCs. The intramuscular implantation of the scaffolds has shown that the immobilization of heparin alone prolongs the acute phase of the inflammatory reaction. However, subsequent treatment with platelet lysate minimizes the inflammatory reaction, slows the rate of implant absorption, and accelerates its vascularization. The results obtained show that the developed bioactive coating improves the cellular properties of PCL electrospun scaffolds and can be useful to form a TEC in vivo. Thus, the obtained scaffold demonstrates the potential for application in tissue-engineered constructs (TECs) within the fields of pharmacology and personalized regenerative medicine.

## Figures and Tables

**Table 1:** Effect of feedrate of 10% PCL solution on the mechanical properties of scaffolds.

Feedrate, mL/h	Young’s Modulus, MPa	Tensile Strength, MPa	Elongation at Break, %
2	3.1 ± 0.3	1.3 ± 0.2	134 ± 9
4	5.5 ± 1.1	2.5 ± 0.4	321 ± 29
6	4.7 ± 0.3	1.9 ± 0.2	298 ± 18
Rat aorta *	1.6 ± 0.4	2.9 ± 0.5	327 ± 38

*—our obtained data.

**Table 2:** Effect of applying bioactive coating on physical and mechanical properties of frames (10% PCL solution, feedrate 4 mL/min).

Feedrate, mL/h	Young’s Modulus, Pa	Tensile Strength, MPa	Maximal Elongation, %
PCL	5.5 ± 1.1	2.0 ± 0.4	321 ± 29
PCL-Hep-PL	4.7 ± 0.4	1.3 ± 0.3	257 ± 20

## Data Availability

The original contributions presented in the study are included in the article, further inquiries can be directed to the corresponding author.

## Abbreviations

The following abbreviations are used in this manuscript:

ECM	Extracellular matrix
TEC	Tissue-engineered constructs
PCL	Polycaprolactone
PLA	Polylactic acid
APTT	Activated partial thromboplastin time
DMEM	Dulbecco’s modified Eagle medium
MSC	Mesenchymal stromal cells
VEGF	Vascular endothelial growth factor
FBGC	Foreign body giant cells
